# Supplementary material for: Exploring Marine-Derived Compounds: In Silico Discovery of Selective Ketohexokinase (KHK) Inhibitors for Metabolic Disease Therapy
Source: Mar Drugs. 2024 Oct 3;22(10):455. doi: 10.3390/md22100455 (PMC11509851; doi:10.3390/md22100455)
Supplement: Supplementary file 1 [file marinedrugs-22-00455-s001.zip › marinedrugs-3220800-supplementary.pdf]

marine drugs

MDPI

Supplementary data

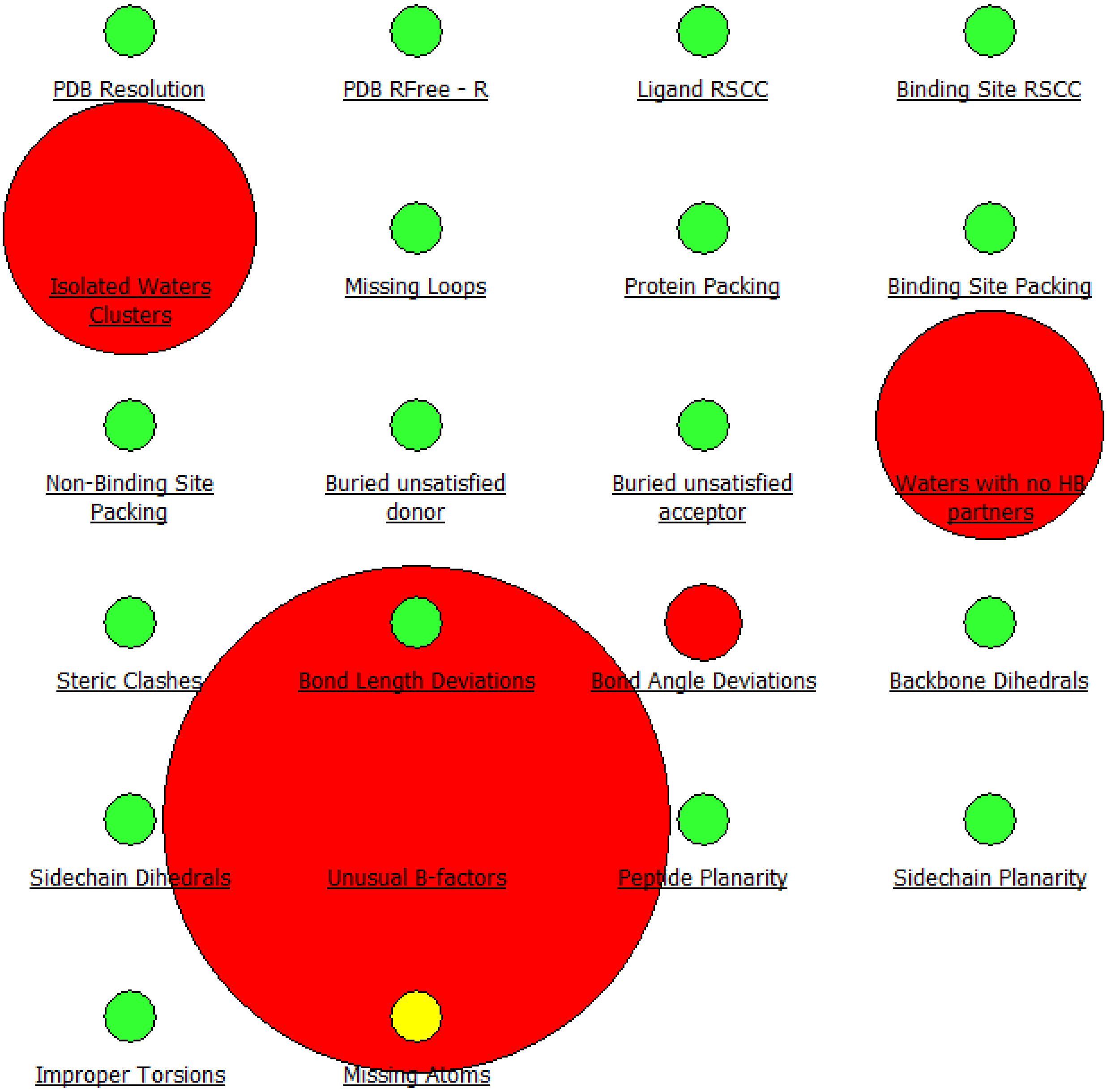

Figure S1. Protein reliability for ketohexokinase.

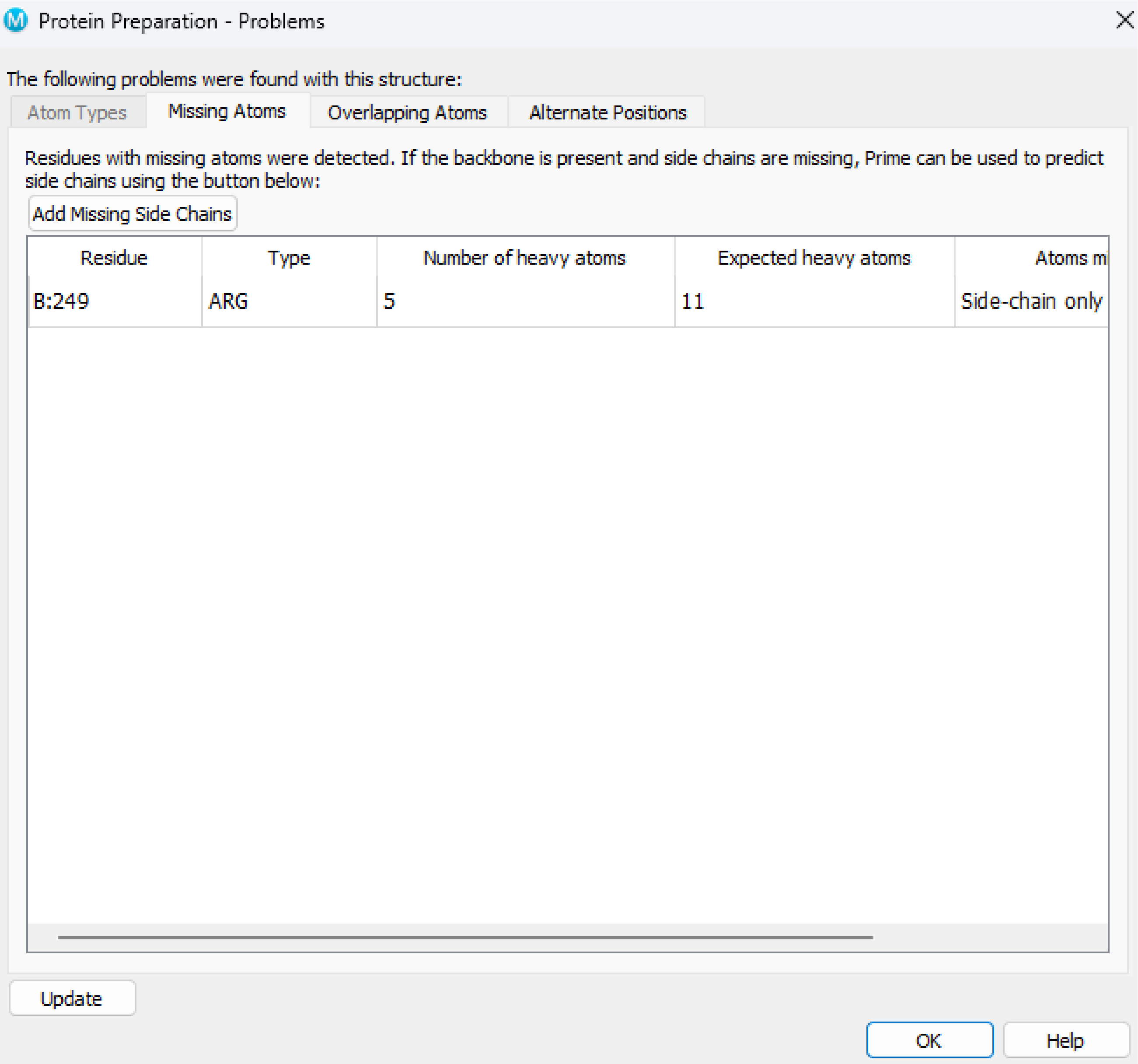

Figure S2. Missing atoms, ARG-249 and several water molecules, including H_2O-485, 487, 493, 495, 572, 579, 580, and 581, lacked hydrogen bond partners.

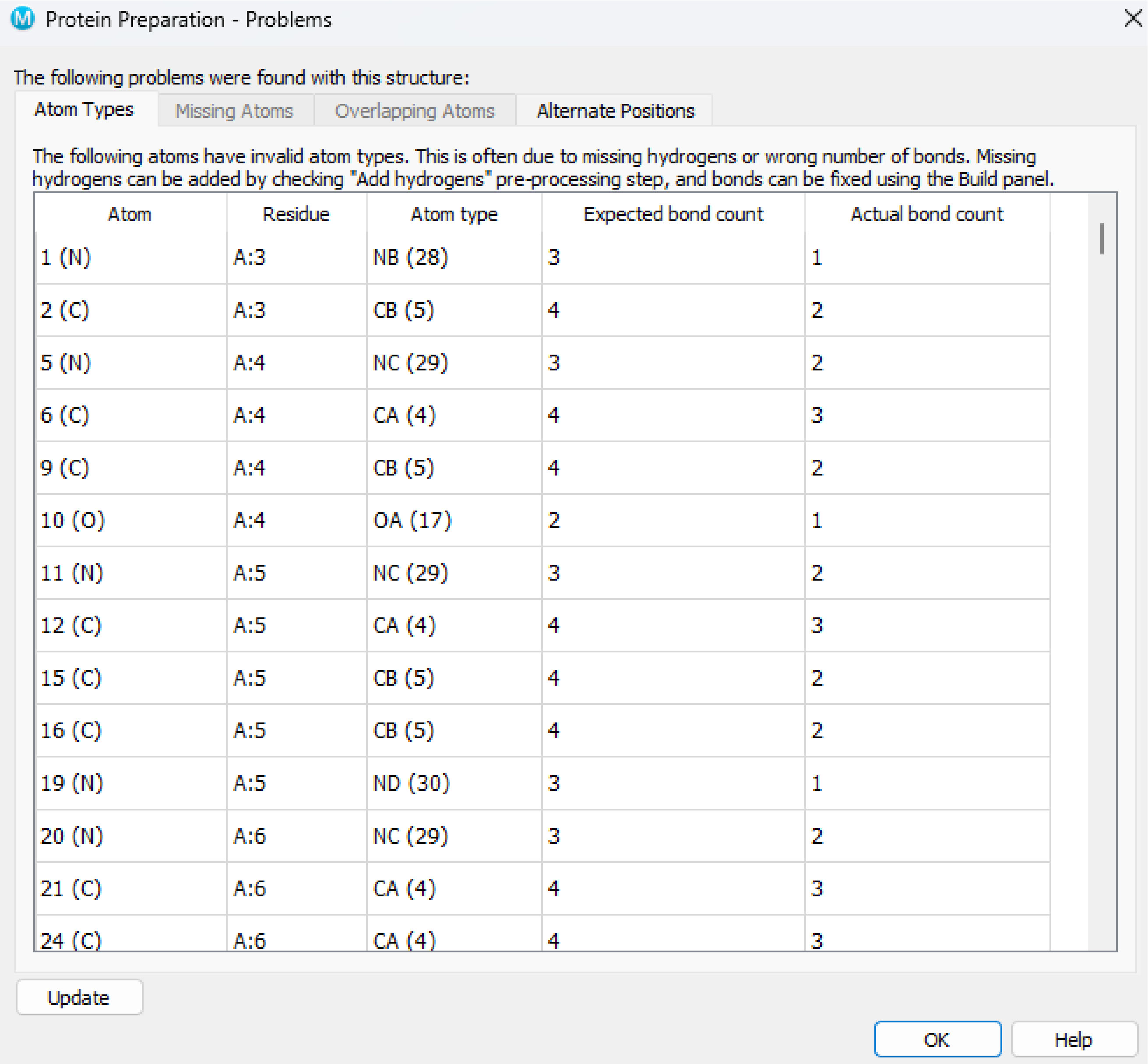

Figure S3. Invalid atom types with missing hydrogen atoms or wrong number of bonds.

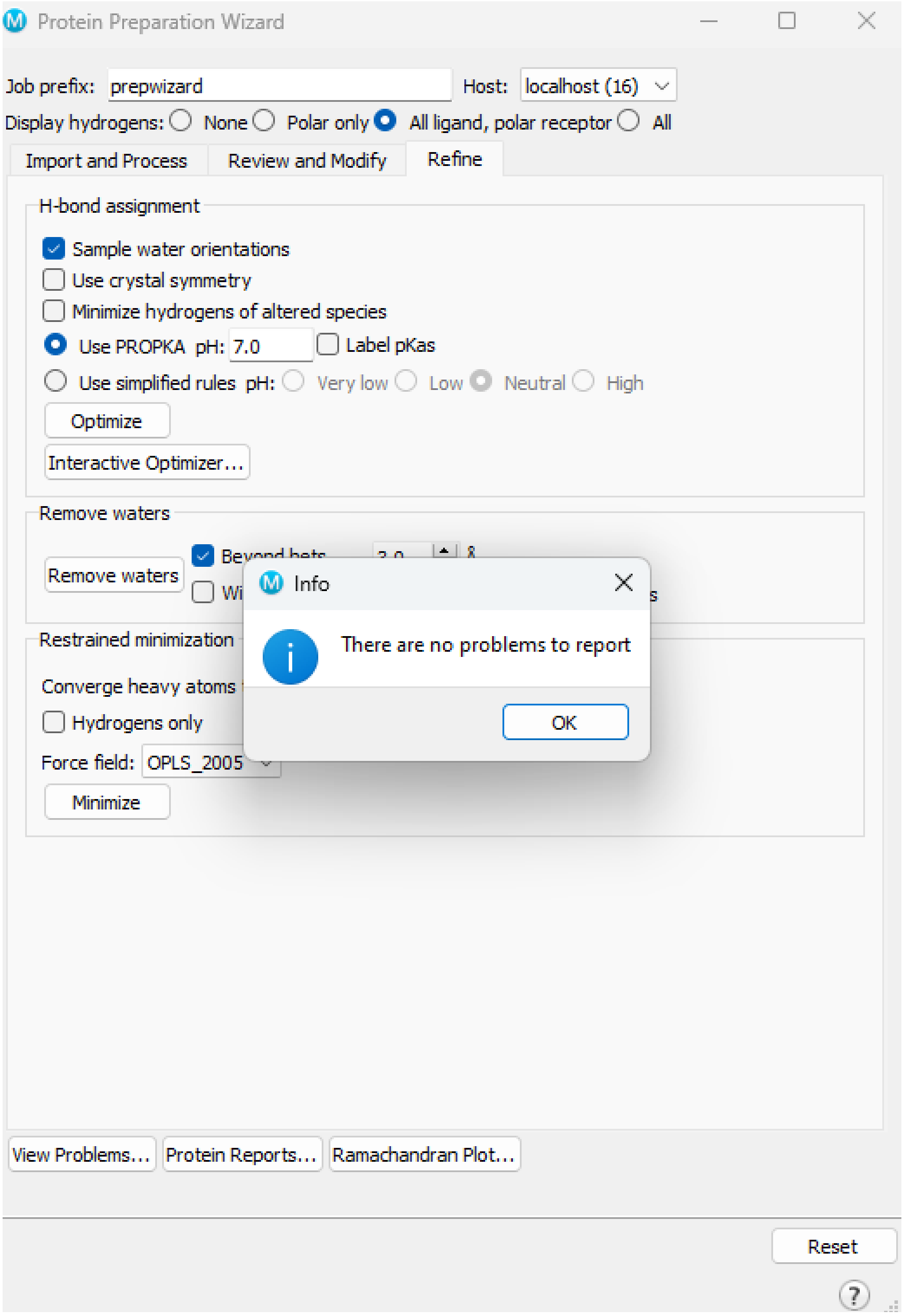

Figure S4. All issues for ketohexokinase crystal structure were addressed, and no further problems were reported.

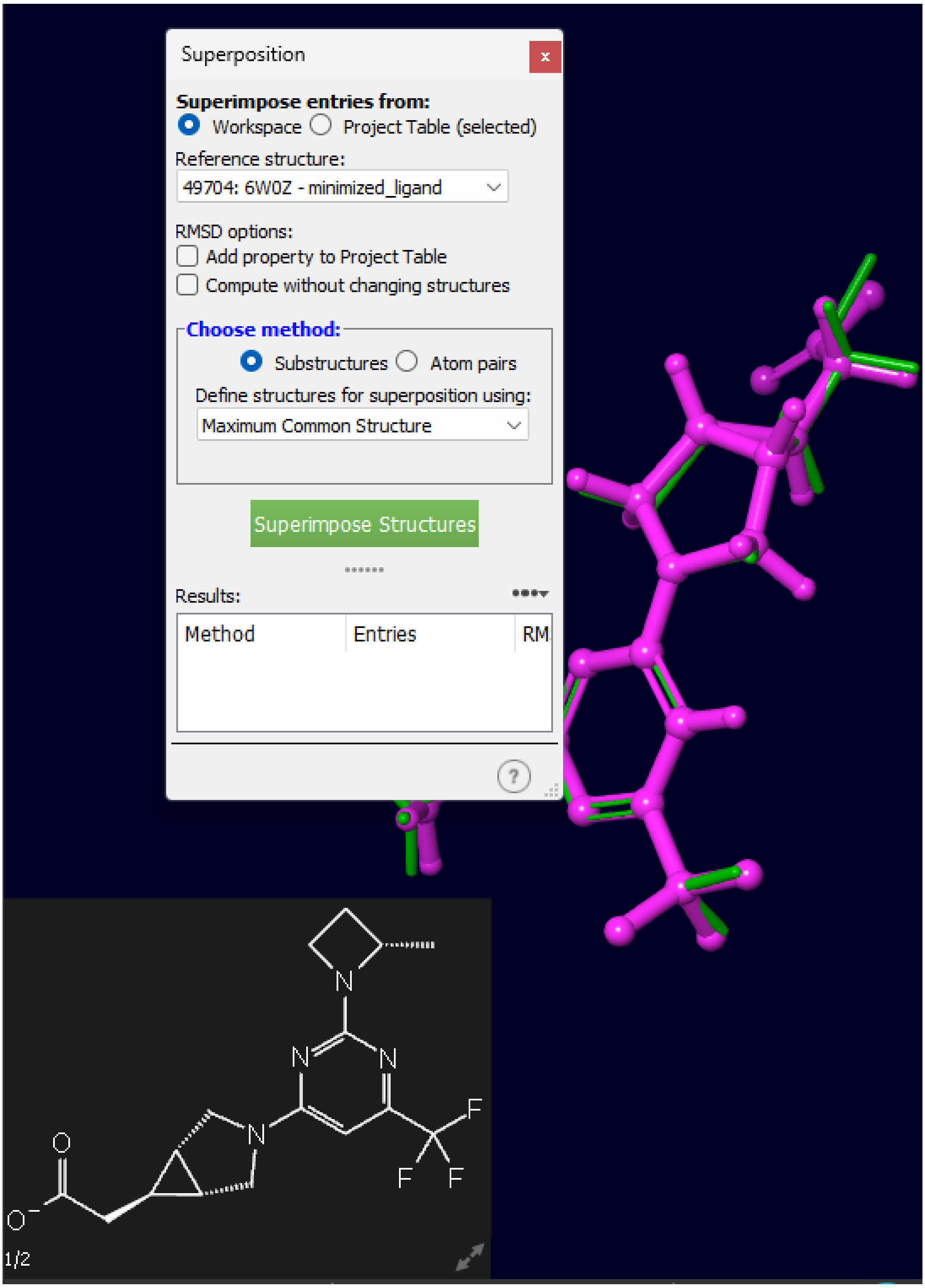

Figure S5. Alignment of the predicted lowest energy conformation of the target with its corresponding co-crystalline ligand.
